# Supplementary material for: A UMLS-based spell checker for natural language processing in vaccine safety
Source: BMC Med Inform Decis Mak. 2007 Feb 12;7:3. doi: 10.1186/1472-6947-7-3 (PMC1805499; doi:10.1186/1472-6947-7-3)
Supplement: Additional file 13 — RAP application directory. Sets up directory for RAP (RDF application for PHP) source code [file 1472-6947-7-3-S13.gz › rap/doc/phpdoc/__filesource/fsource_rdql__rdqlRdqlResultIterator.php.html]

xml version="1.0" encoding="iso-8859-1"?


File Source for RdqlResultIterator.php


# Source for file RdqlResultIterator.php

Documentation is available at RdqlResultIterator.php

```
1. <?php

3. // ----------------------------------------------------------------------------------
4. // Class: RdqlResultIterator
5. // ----------------------------------------------------------------------------------


9. /**
10. * Iterator for traversing Rdql results.
11. * This class can be used for iterating forward and backward trough Rdql results.
12. * It should be instanced using the rdqlQueryAsIterator() method of a MemModel or a DBModel.
13. *
14. *
15. * <BR><BR>History:<UL>
16. * <LI>25-03-2004                 : Bug in the handling of empty results fixed (radol@gmx.de).</LI>
17. * <LI>11-06-2003                : First version of this class.</LI>
18. * </UL>
19. *
20. * @version  V0.9.1
21. * @author Daniel Westphal <mail@d-westphal.de>, Chris Bizer <chris@bizer.de>
22. *
23. * @package rdql
24. * @access    public
25. *
26. */
27. class RdqlResultIterator extends Object {

29. /**
30. * Reference to the Rdql result
31. * @var        array RdqlResult
32. * @access    private
33. */
34. var $RdqlResult;

36. /**
37. * Current position
38. * RdqlResultIterator does not use the build in PHP array iterator,
39. * so you can use serveral iterators on a single Rdql result.
40. * @var        integer
41. * @access    private
42. */
43. var $position;


46. /**
47. * Constructor
48. *
49. * @param    object RdqlResult
50. * @access    public
51. */
52. function RdqlResultIterator(&$RdqlResult) {

54. $noResult = TRUE;
55. foreach($RdqlResult[0] as $value)
56. if ($value != NULL) {
57. $noResult = FALSE;
58. break;
59. }

61. if($noResult)
62. $this->RdqlResult = NULL;
63. else
64. $this->RdqlResult = $RdqlResult;

66. $this->position = -1;
67. }

69. /**
70. * Returns the labels of the result as array.
71. * @return    array of strings with the result labels OR null if there are no results.
72. * @access    public
73. */
74. function getResultLabels() {
75. if(count($this->RdqlResult)>0) {
76. return array_keys($this->RdqlResult[0]);
77. } else return null;
78. }

80. /**
81. * Returns the number of results.
82. * @return    integer
83. * @access    public
84. */
85. function countResults() {

87. return count($this->RdqlResult);

89. }
90. /**
91. * Returns TRUE if there are more results.
92. * @return    boolean
93. * @access    public
94. */
95. function hasNext() {
96. if ($this->position < count($this->RdqlResult) - 1 ) {
97. return TRUE;
98. } else {
99. return FALSE;
100. }
101. }

103. /**
104. * Returns TRUE if the first result has not been reached.
105. * @return    boolean
106. * @access    public
107. */
108. function hasPrevious() {
109. if ($this->position > 0) {
110. return TRUE;
111. } else {
112. return FALSE;
113. }   }

115. /**
116. * Returns the next result array.
117. * @param     integer $element
118. * @return    result array OR single result if $element was specified OR NULL if there is no next result.
119. * @access    public
120. */
121. function next($element = null) {
122. if ($this->position < count($this->RdqlResult) - 1) {
123. $this->position++;
124. if ($element) {return $this->RdqlResult[$this->position][$element];}
125. else return $this->RdqlResult[$this->position];
126. } else {
127. return NULL;
128. }
129. }

131. /**
132. * Returns the previous result.
133. * @param     integer $element
134. * @return    result array OR single result if $element was specified OR NULL if there is no next result.
135. * @access    public
136. */
137. function previous($element = null) {
138. if ($this->position > 0) {
139. $this->position--;
140. if ($element) {return $this->RdqlResult[$this->position][$element];}
141. else return $this->RdqlResult[$this->position];
142. } else {
143. return NULL;
144. }
145. }

147. /**
148. * Returns the current result.
149. * @param     integer $element
150. * @return    result array OR single result if $element was specified OR NULL if there is no next result.
151. * @access    public
152. */
153. function current($element = null) {
154. if (($this->position >= 0) && ($this->position < count($this->RdqlResult))) {
155. if ($element) {return $this->RdqlResult[$this->position][$element];}
156. else return $this->RdqlResult[$this->position];
157. } else {
158. return NULL;
159. }
160. }

162. /**
163. * Moves the pointer to the first result.
164. * @return    void
165. * @access    public
166. */
167. function moveFirst() {
168. $this->position = 0;
169. }

171. /**
172. * Moves the pointer to the last result.
173. * @return    void
174. * @access    public
175. */
176. function moveLast() {
177. $this->position = count($this->RdqlResult) - 1;
178. }

180. /**
181. * Moves the pointer to a specific result.
182. * If you set an off-bounds value, next(), previous() and current() will return NULL
183. * @return    void
184. * @access    public
185. */
186. function moveTo($position) {
187. $this->position = $position;
188. }

190. /**
191. * Returns the current position of the iterator.
192. * @return    integer
193. * @access    public
194. */
195. function getCurrentPosition() {
196. return $this->position;
197. }

199. }

201. ?>
```

Documentation generated on Thu, 7 Jul 2005 13:43:28 +0200 by phpDocumentor 1.3.0RC3
